# The Global Problem of Insufficient Sleep and Its Serious Public Health Implications

**DOI:** 10.3390/healthcare7010001

**Published:** 2018-12-20

**Authors:** Vijay Kumar Chattu, Md. Dilshad Manzar, Soosanna Kumary, Deepa Burman, David Warren Spence, Seithikurippu R. Pandi-Perumal

**Affiliations:** 1 Faculty of Medical Sciences, The University of the West Indies, St. Augustine, Trinidad and Tobago; susanna.poul@gmail.com; 2 Department of Nursing, College of Applied Medical Sciences, Majmaah University, Majmaah 11952, Saudi Arabia; m.manzar@mu.edu.sa; 3 School of Medicine, University of Pittsburgh, 4200 Fifth Ave, Pittsburgh, PA 15260, USA; dr.deepa.burman@gmail.com; 4 Independent Researcher, 652 Dufferin Street, Toronto, ON M6K 2B4, Canada; dwspence@fastmail.fm; 5 Somnogen Canada Inc., College Street, Toronto, ON M1H 1C5, Canada; pandiperumal2019@gmail.com

**Keywords:** insufficient sleep, insomnia, sleep disorders, sleep apnoea, cardiometabolic diseases

## Abstract

Good sleep is necessary for good physical and mental health and a good quality of life. Insufficient sleep is a pervasive and prominent problem in the modern 24-h society. A considerable body of evidence suggests that insufficient sleep causes hosts of adverse medical and mental dysfunctions. An extensive literature search was done in all the major databases for “insufficient sleep” and “public health implications” in this review. Globally, insufficient sleep is prevalent across various age groups, considered to be a public health epidemic that is often unrecognized, under-reported, and that has rather high economic costs. This paper addresses a brief overview on insufficient sleep, causes, and consequences, and how it adds to the existing burden of diseases. Insufficient sleep leads to the derailment of body systems, leading to increased incidences of cardiovascular morbidity, increased chances of diabetes mellitus, obesity, derailment of cognitive functions, vehicular accidents, and increased accidents at workplaces. The increased usage of smart phones and electronic devices is worsening the epidemic. Adolescents with insufficient sleep are likely to be overweight and may suffer from depressive symptoms. The paper concludes by emphasizing sleep quality assessments as an important early risk indicator, thereby reducing the incidence of a wide spectrum of morbidities.

## 1. Introduction

Insufficient sleep is associated with a range of negative health and social outcomes, including an adverse performance at school and in the labor market. Reduced sleep duration has been linked to 7 of the 15 leading causes of death in the U.S., including cardiovascular disease, malignant neoplasm, cerebrovascular disease, accidents, diabetes, septicemia, and hypertension [1]. The evidence suggests that the link between inadequate sleep and negative outcomes is more direct, whereas the link between excessive sleep and negative outcomes seems to be more indirect (i.e., excessive sleep is driven by underlying chronic health conditions and not vice versa). Hence, the impact of insufficient sleep appears to be the more salient issue in our society and, because of its broad-ranging effects, represents a major public health concern. In this review, we note some of the more serious consequences of insufficient sleep, and additionally consider how these might be best addressed by changes in individual behavior, actions by employers, and by public policy measures [2].

According to the International Classification of Sleep Disorders (ICSD-3), insufficient sleep is defined as a curtailed sleep pattern that has persisted for at least three months for most days of the week, along with complaints of sleepiness during the day. Further, a resolution of sleepiness complaints is shown to follow an extension of total sleep time. Frequently occurring episodes of insufficient sleep are associated with the experience of unfavorable mental and physical well-being [3]. Sleep insufficiency is sometimes confused with insomnia, but the opportunity to sleep differs in the two disorders (with insomnia sufferers typically being unable to sleep despite having opportunities to do so).

## 2. Materials and Methods

**Figure 1:**
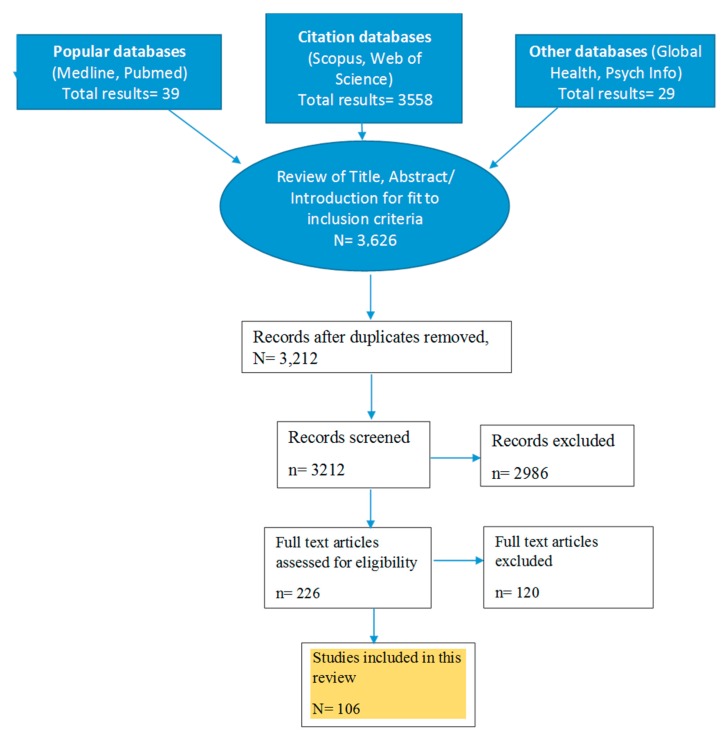
Flow chart of literature search.

An extensive literature search was done, and relevant articles were identified through online searches of electronic databases (i.e., the PubMed, Medline, PsycINFO, Web of Science, Scopus, and Global Health databases). Relevant keywords relating to “insufficient sleep”, in combination with (and/or) public health impact were searched, around 3626 articles were screened for duplication and relevance to fit the inclusion criteria, and finally 111 articles with full texts were included in this review, as shown in the flow chart below (Figure 1). Articles on cardiometabolic disorders, mental health, accidents and trauma, sleep apnoea, obesity, adolescents, meta-analysis, randomized control trials, longitudinal studies, and cross-sectional studies were included (that reflected the public health impacts of insufficient sleep). Additional publications were identified from references cited in the original articles. The major findings were classified into different categories and are presented in the tables, figures, and narrative description below.

### 2.1. Epidemiology

A recent study of the prevalence of sleep disorders investigated over 20,000 patients in the Netherlands who were aged 12 years old or older. The study found an alarming prevalence rate of 27.3%, with 21.2% of the males and 33.2% of the females reporting that they had some type of sleep disorder [4]. A national quasirandom sample study of sleep disorders in 12 provinces of the Netherlands showed a prevalence of 23.5% [5], while a similar nationally representative sample of individuals in the age group of 18–70 years also showed high prevalence rates. Polling based on a validated questionnaire of ICSD revealed that, among the total sample, 32% complained of experiencing general sleep disturbances while 43.2% said they suffered from insufficient sleep [6]. Other surveys by Hublin et al. [7] and Ursin et al. [8] found the prevalence rates for disturbed sleep to be, respectively, 20.4% and 20%. Discrepancies in the prevalence rates found by the various surveys were possibly due to the separate questionnaires that were used, differences in sampling techniques, or other possible methodological differences. Nevertheless, the findings were in concordance in showing that poor-quality sleep is a problem for a major segment of the samples that were surveyed.

Although the need for sleep among adolescents has been determined to be about 9.25 h per day [9], there are still many adolescents who obtain less sleep than they actually need [10], thus creating a chronic sleep debt. A study conducted in Norway among 1285 high school students (aged 16–19 years) showed an estimated 10.4% prevalence of behaviorally induced insufficient sleep syndrome (i.e., the condition was due to individual choices regarding bedtimes rather than being the consequence of a medical condition) [11]. Among a sample of 2738 soldiers who were surveyed about their sleep behavior and perceived feelings of sleepiness, the average reported sleep duration was found to be 5.8 ± 1.2 h, with 1959 soldiers, or 72% of the sample, reporting that they slept less than 6 h [12].

Sleep needs vary among individuals based on age, and even response to sleep restriction changes with age. According to a Centers for Disease Control and Prevention (CDC) state-based survey in 2014, only 65% of adults reported a healthy duration of sleep. In a recent survey, an estimated 83.6 million adults in the United States were reportedly sleeping <7 h in 24 h [13]. The prevalence of continuous insufficient sleep among American Indians and Alaska Natives (AI/AN) has not been formally surveyed, but anecdotal reports suggest that the figure is high in these minority groups. It has been suggested that the rates of mental disturbance that are also known to be elevated in these groups may possibly be, in part, attributable to insufficient sleep, and thus that public health and education efforts to address these problems should be undertaken [14].

**Figure 2:**
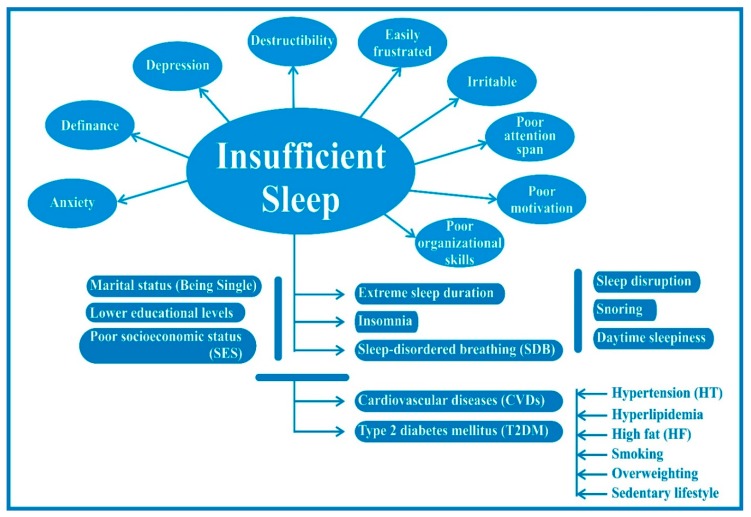
Insufficient sleep and its impact on the pathophysiology of the human body.

Magee et al. [15], for instance, explored the determinants of sleep duration among a cohort of Australian adults aged 18 to 64 years. Using multivariate statistical analysis, the study found that short sleep durations were associated with longer working hours, lower education levels, being single rather than married, being a current cigarette smoker, or with showing high levels of alcohol consumption, obesity, or depression or anxiety, as shown in Figure 2 below. Krueger and Friedman [16] found that factors such as low levels of education and cardiovascular disease were associated with both short and long sleep (e.g., more than nine hours). Short sleep was associated with being in an older age group, being a frequent smoker or consumer of alcohol, being overweight or obese, as well as with having young children. A study from Finland by Kronholm et al. [17] reported that gender, as well as marital status, occupation, and physical activity, were major drivers of short sleep duration. According to the study, men were more likely to be short sleepers than women. A study of the effects of workplace-related factors found that job stress factors such as quantitative workload and interpersonal conflict led to short sleep duration among male Japanese manufacturing workers [18]. Psychosocial factors such as tension or anxiety and depressive symptoms have also been associated with reduced sleep. In addition, organizational factors related to discrimination, work-life balance, high work demands, and job insecurity were associated with an increased prevalence of sleep problems [19,20].

Lack of sleep has been shown to increase the risk of premature mortality. In a recent review of various surveys, it was concluded that individuals who slept for less than six hours each night had a tenfold greater risk of premature mortality than those who obtained seven to nine hours of sleep [21]. Given the potential antagonistic impacts of inadequate mull or consideration over well-being, prosperity, and profitability, the outcomes of lack of sleep have expensive financial results. Insufficient sleep is a global problem that is becoming increasingly common in today’s society. Compared to a few decades ago, significant changes in sleep culture have been observed worldwide. This global trend has produced massive social and economic shifts, and additionally has had marked public health consequences, and foremost among these is the significant reduction in total sleeping hours that have occurred in both adults and children [22].

### 2.2. All-Cause Mortality

Epidemiological evidence suggests that sleep duration and poor sleep are associated with premature mortality, as well as with an extensive variety of adverse health outcomes [23]. The Sleep Heart Study was done to determine the association between insufficient sleep conditions, including insomnia or poor-quality sleep and objectively measured short duration sleep, and the incidence of cardiovascular disease (CVD) and mortality in the general population. The study found that there was a 29% higher risk of CVD in study subjects who had insomnia or poor sleep with short sleep when compared to a reference group, thus supporting the conclusion that poor sleep with objectively measured short sleep was associated with a higher risk of CVD development [24].

### 2.3. Sleep Duration Recommendations by Age

The American Academy of Sleep Medicine (AASM) and the Sleep Research Society (SRS) have recommended that adults aged 18 to 60 years should sleep seven or more hours per night on a regular basis for ideal sleep health. Additionally, the National Sleep Foundation (NSF) consensus report has stated that seven to nine hours is recommended for adults aged 18 to 64 years, while seven to eight hours is suggested for those 65 years of age and older [25].

### 2.4. Pathophysiology

There are specific biomarkers that are released throughout insufficient sleep. Experimental studies with human subjects have demonstrated that proinflammatory markers undergo changes following laboratory-induced sleep loss. Markers that have been found to be dysregulated in acute sleep deficiency include IL-6 [26], IL1 receptor antagonist [27], and salivary amylase. However, there are biomarkers needed for the prediction of sleepiness and other consequences of sleep deprivation. Another study used NMR measures of metabolism to determine how the pathways involved in cholesterol metabolism and inflammatory responses changed due to prolonged sleep restriction. The investigation found that, compared to normal, sleep-restricted subjects showed decreases in levels of low-density lipoprotein (LDL), whereas there were no significant changes in high-density lipoprotein (HDL) levels. In a comparison group of subjects who reported that they suffered from subjective sleep insufficiency, HDL levels were decreased, but LDL levels did not differ from those of the restricted group [28]. In an analysis of data from 1024 adults surveyed in the Wisconsin Sleep Cohort Study, it was found that short sleep durations were associated with lower leptin and higher ghrelin levels [29].

### 2.5. Contributing Factors

Studies have observed that sleep duration and daytime sleepiness varies by gender and marital status. The presence of children in a family often contributes to insufficient rest or sleep among the adults with whom they live [14]. Insufficient sleep is typically a long-standing condition, often linked to biological or circadian disruption factors. Reviews of studies of possible causal factors have additionally suggested that genetic influences may account for possibly as much as one-third of cases of insufficient sleep [7]. Insufficient sleep duration is associated with an elevated intake of soda via excessive consumption of salt or carbonated beverages, and less frequent vegetable consumption [30]. Various types of stress also contribute. Housing insecurity and food uncertainty are psychological stressors associated with insufficient sleep [31]. Social and behavioral predictors of health exclusively add to the explosion of insufficient sleep [32]. Active smoking, smokeless tobacco, and secondhand smoke exposure have been additionally associated with insufficient sleep [33]. An increasing number of studies have shown that insufficient sleep and behavioral or emotional problems have reciprocal and mutually facilitating effects. It is known, for instance, that mood disorders are frequently associated with severe sleep problems and that the problems that are linked to inadequate sleep, such as reduced impulse control, reductions in attention span, and memory impairments, may further exacerbate a pre-existing mood disorder [34].

## 3. Results: Manifestations of Insufficient Sleep

### 3.1. Cognitive Effects

Insufficient sleep can contribute to aberrant behavior. Subjects who are chronically sleep-restricted may exhibit increased risk-taking behavior, or subjectively may show deficiencies in reasoning that result from seeking premature conclusions without considering all aspects of a problem. This type of impulsivity may manifest also as increased but unnoticed risk-seeking [35]. Insufficient sleep that occurs in children in the preschool and early school years is associated with poorer mother- and teacher-reported neurobehavioral processes, which particularly manifest in mid-childhood [36].

### 3.2. Mood and Judgment

Chronic sleep restriction among adolescents may increase suicidal risk [37]. Sleep loss can have adverse effects on the control of mood and behavior. Irritability, moodiness, and poor frustration tolerance are the most frequently described symptoms in subjects who are suffering from sleep restriction [34]. One study investigated the behavioral and psychological consequences of chronic sleep deprivation in different age or social groups. Among all groups, sleep restriction had generally adverse effects, but some subgroup differences were noted. Adolescents complained of tiredness upon awakening (46%), nervousness, and general weakness; university students reported experiencing excessive drowsiness (50%), tension, and nervousness; and working adults suffered mostly from negative moods, such as tension (49%), nervousness, and irritability [38].

### 3.3. Sleepiness and Microsleep

Daytime sleepiness is the most direct result of sleep loss reported by adolescents and manifests most significantly as difficulty in getting up in the morning. Sleepiness is also connected to a strong tendency toward brief mental lapses (or microsleep episodes), occurrences that significantly increase the risk of motor vehicle and other accidents [34]. A study based on the monitoring of microsleep episodes was carried out to investigate the consequences of sleep restriction (SR) on the maintenance of wakefulness and diurnal sleepiness. The investigators found that during sleep restriction, daytime microsleeping correlated with the Karolinska Sleepiness Scale (KSS) but not with the Maintenance of Wakefulness Test (MWT). The study also concluded that the frequency of microsleeping is an objective marker of diurnal sleep pressure [39].

### 3.4. Effects on Respiratory Physiology

There is growing interest in the impact of sleep and its disorders on the regulation of inflammatory processes and tissue morbidities, particularly in the context of metabolic and cardiovascular diseases. Obstructive sleep apnea syndrome (OSAS) is commonly seen in conjunction with insufficient sleep [40].

The condition of narcolepsy-cataplexy is characterized by disrupted nocturnal sleep as one of its most noticeable features, which in turn contributes to excessive daytime sleepiness in affected patients. Another feature of the condition is the lack of adequate non-rapid eye movement (NREM) sleep, which is related to nonconsolidated nocturnal sleep in narcolepsy-cataplexy, which also occurs in this patient group [41].

### 3.5. Wakefulness and Vigilance

Chronic insufficient sleep duration equivalent to an average of 5.6 h of sleep during a 24-h period has been found to double neurobehavioral reaction time performance and to increase lapses of attention fivefold. Impairments in neurobehavioral performance were worsened during the circadian night and did not recover during the circadian day, thus indicating that the deleterious effect from the homeostatic buildup of chronic sleep restriction (CSR) is expressed even during the circadian promotion of daytime arousal [42].

### 3.6. Tiredness and Fatigue

There is evidence that sleep loss can impair active cognitive processes such as planning, coping, and problem-solving. In sleep-impaired individuals, these deficits may particularly manifest in behaviors that require creative solutions to problems that are either complex or are lacking in sufficient information. The energy that is required to analyze unfamiliar environmental challenges or to sustain an extended chain of logical thought is especially reduced in sleep-deprived subjects. This internal state cognitive impairment may thus affect their ability to initiate behaviors related to long-term or abstract goals, and, as a result, may decrease their motivation to work toward those goals [34].

### 3.7. Effects on Mental Health

Chronic sleep loss and associated sleepiness and daytime impairments in adolescence are a genuine impediment to the achievement of academic success, health (for example depression, increased obesity risk), and safety (such as driving accidents) [43]. Emotional excitement and pain can cause difficulties with either sleep initiation or sleep maintenance. Behavioral issues and family conflict can contribute to even later bedtimes and to sleep schedules that are particularly at variance with daily schedules [34]. Further, a number of studies have shown that inadequate sleep increases the likelihood of daytime accidents and critical mistakes in the workplace.

### 3.8. Increased Incidence of Cardiovascular Morbidity

Insufficient sleep is associated with cardiovascular disease, and has been studied in numerous racial and ethnic groups. Similarly, the association between insufficient sleep and diabetes mellitus has been demonstrated to occur in a number of racial and ethnic minorities, with the exception of non-Hispanic blacks [44]. Insufficient sleep is also associated with cardiometabolic risk and neurocognitive impairment. Determinants of insufficient sleep include many social and environmental factors. Surveys have shown that the incidence of inadequate sleep is not consistent across all areas of the United States. Respondents in a few areas, and particularly in Appalachia, have reported excessively high levels of inadequate rest [45]. Insufficient sleep has also been shown to be linked to an increased risk of acute myocardial infarction [46]. It has been reported by Curtis [47] that more than one-half of racial differences in cardiometabolic risk can actually be explained by sleep patterns, and, more specifically, that, compared to Caucasian American adults, the reduced total sleep time and lower sleep efficiency of African-Americans is largely attributable to lifestyle or personal choice factors rather than racial or genetic influences. The study found that, compared to Caucasian Americans, African Americans obtained less sleep (341 vs. 381 min) and had lower sleep efficiency (72.3 vs. 82.2%) (*P-*value < 0.001). Further, 41% and 58% of the racial difference in cardiometabolic risk was explained by sleep time and sleep efficiency, respectively.

### 3.9. Effects on the Immune System

Sleep loss can adversely affect components of the immune system critical to host resistance to infectious illness. Furthermore, short sleep duration and sleep disturbances prospectively predict increased susceptibility to upper respiratory tract infection after an experimental viral challenge [48]. Additional findings are listed in Table 1.

### 3.10. Obesity and Metabolism

Sleep plays a pivotal role in energy metabolism. Both sufficient sleep and high-quality diets are vital for the prevention of obesity [49]. Various studies have shown linkages between insufficient sleep and obesity. Increased food intake during insufficient sleep is a physiological adaptation to provide the energy needed to sustain additional wakefulness, yet when food is easily accessible, intake often exceeds that which is required [50]. There is reliable evidence supporting the conclusion that sleep restriction increases food intake, and further that this association is due to increased production of the appetitive hormone ghrelin [29,51]. As a consequence, insufficient sleep is associated with increases in body mass index (BMI). Sleep loss can alter energy intake and expenditure. The amount of human sleep contributes to the maintenance of fat-free body mass at times of decreased energy intake. Lack of sufficient sleep may compromise dietary interventions for weight loss and related metabolic risk reduction [52]. Chronic sleep restriction is a potential risk factor for the maintenance of metabolic health. There is an association between insufficient sleep and an increased incidence of obesity and related morbidities. Thus, overweight and obese individuals attempting to reduce their caloric intake and maintain increased physical activity should obtain adequate sleep and, if needed, seek effective treatment for any coexisting sleep disorders [53].

### 3.11. Increased Risk for Diabetes Mellitus

Insufficient sleep, or more precisely the suppression of slow wave sleep and rapid eye movement sleep, has been associated with insulin resistance [54]. There is a high prevalence of insufficient sleep in young patients with diabetes mellitus type 1 (type 1 diabetes) and their relatives [55]. Inadequate sleep is also associated with an increased risk of hypertension, cardiovascular disease, and mortality. Lower scores on a scale of self-rated health (SRH), which are also linked to insufficient sleep, have been shown to indicate an elevated risk of CVD and death [56].

### 3.12. Migraine

The prevalence of insufficient sleep is significantly higher in patients with migraines when compared to those in nonmigraine headache or nonheadache groups [57]. The Chronic Migraine Epidemiology and Outcomes (CaMEO) Study, which assessed the relationship between sleep disturbances, including sleep apnea, and episodic or chronic migraines, concluded that the conditions were associated, and that the risk was enhanced in the elderly and in those with a higher BMI [58].

### 3.13. Clinical Burnout

In one study, it was found that insufficient sleep, preoccupation with thoughts of work during leisure time, and high work demands were risk factors for consequent clinical burnout [59]. It has also been found that sleep problems tend to precede symptoms of low back pain (LBP) and burnout in working individuals. It has therefore been suggested that health promotion initiatives should emphasize assessments of sleep quality as an important early risk indicator, and, further, that interventions should focus on promoting a better quality of sleep, in an attempt to reduce the incidence of LBP and burnout [60].

### 3.14. Increased Risk of Cancers

Insufficient sleep, including short sleep duration and sleep disruption, might be related to an increased risk of cancerous tumor formation [61]. Sleep disturbance increases the risk of breast cancer. It has been suggested that melatonin is involved in the relationship between sleep and breast cancer, in as much as exogenously applied melatonin provides an antiproliferative effect on breast cancer cells [62].

### 3.15. Sleep-Wake Disturbances in Shift Workers

Self-reported bruxism may indicate sleep problems and associated fatigue in daytime waking hours in nonpatient populations. It may be more prevalent among people who do shift work schedules or who work in jobs with irregular hours [63]. Laboratory studies have indicated that cardiometabolic stress and cognitive impairments are increased by shift work, as well as by insufficient sleep. A recent study by Kervezee [64] found that four days of simulated night shifts was associated with a loss in temporal coordination between internal circadian rhythmicity and the external environment, which in turn produced broader associated adverse health effects, which are commonly seen in night shift workers. A recent review [65] based on 38 meta-analyses and 24 systematic reviews summarized the linkages between shift work and adverse consequences, such as insufficient sleep, cardiovascular diseases, and accidents. The meta-analysis showed that shift work was closely linked to cardiometabolic diseases and accidents and that these associations tended to mimic those seen in persons with insufficient sleep. All these impairments because of insufficient sleep are described in Table 1 below.

## 4. Discussion

The general public often devalues the seriousness of insufficient sleep and may have a more general attitude that, in the larger scheme of life’s difficulties, “not getting enough sleep” occupies a fairly low rung on the stepladder of personal health concerns. As a consequence, sleep insufficiency often goes unreported by patients in medical interviews. Some senior specialists skillfully and intentionally deal with the issue of their patients’ sleeping habits, but, as the evidence of the studies reviewed here show, the significance of sleep to health status deserves particular and earnest attention.

To ensure that they obtain sufficient sleep, patients should set predictable wake-up times, refrain from using electronic devices prior to their normal sleep time, and obtain adequate physical exercise [21]. Employers need to be made aware of the significance of rest for overall health and the responsibility of businesses to provide a work environment and conditions that will not interfere with an employee’s right to adequate sleep. Business management needs to plan for and create brighter workspaces. Business managers should also strive to discourage conflict in the workplace and discourage the use of electronic devices. Further, school officials should consider establishing later school start times to avoid interference with students’ critical need for sleep, which exists during the adolescent years [21].

Policymakers should encourage educational efforts to raise awareness about the importance of sleep, and especially emphasize the contribution that employers can make to ensuring that work assignments do not adversely interfere with sleep schedules. It has been found that adolescents who do not obtain sufficient sleep are likely to be overweight due to lack of engagement in daily physical activity, may suffer from depressive symptoms, may engage in risky behaviors (drinking, smoking tobacco, and using illicit drugs), and may perform inadequately in school [106].

## 5. Conclusions

Insufficient sleep and its health consequences may go unrecognized by clinicians inasmuch as many medical school curriculums do not emphasize the importance of sleep to overall health. The causes of common patient complaints of daytime weakness, tiredness, sluggishness, languid driving, and intellectual troubles may often be misattributed to life stresses such as family or social problems rather than to the more basic cause of inadequate rest. There is a clear need for medical professionals to ensure that their patients are made aware of common contributors to sleep disruption such as jet lag or shift work. However, more fundamentally, patient attitudes about the adverse effects of inadequate sleep for health need to be addressed.

## Figures and Tables

**Table 1:** Sleep deprivation, which has been recognized as a “public health epidemic” linked to a range of medical and mental health issues.

Health Issues and Impairments Associated with Sleep Deprivation	Comments	References
Hypertension	Sleep deprivation is linked to an increased risk of hypertension	Schlafer et al., 2014 [66]
Cardiovascular incidents	Sleep-deprived people are at greater risk for coronary heart disease	Ayas et al., 2003 [67]
	Increased heart attacks	Janszky and Ljung, 2008 [68]
Type 2 diabetes mellitus (T2DM)	Sleep deprivation is linked to an increased risk of hypertension	Schlafer et al., 2014 [66]
Depression	Increases the risk of developing depression	Chen et al., 2012 [69]
	Increases the relapse of depression	Howland, 2011 [70]; Okun et al., 2011 [71]
	High reporting of depressive symptoms among students	O’Brien, 2005 [72]; Catrett, 2009 [73]
	Anxiety, depression, and withdrawal increased depression among adolescents	Coulombe et al., 2010 [74]; Pallesen et al.,2011 [11]; Lund et al., 2010 [75]; Roberts et al., 2009 [76]
Obesity	Sleep deprivation is linked to an increased risk of obesity	Schlafer et al., 2014 [66]
	Suffer more obesity	Taheri et al., 2004 [29]
	Increased obesity among adolescents by 80%	Gupta et al., 2002 [77]
Cancer	Linked to an increased risk of cancer	Markt S.C. et al., 2015 [61]; Lehrer S. et al., 2013 [62]
Mortality risks	Die at an early age	Kripe et al., 2002 [78]
Cognitive performance	Impairs visuomotor performance	Paula Alhola and Paivi Kantola, 2007 [79]
	Impairment in saccadic eye movements	Bocca and Denise, 2006 [80]
	Increases rigid thinking, perseveration errors, and difficulties in utilizing new information in complex tasks	Harrison and Horne, 1999 [81]
	Affects high-level cognitive executive functions	Beebe, 2011 [82]
Memory	Impaired performance in probed force memory recall and memory search	Wright and Badia, 1999 [83] McCarthy and Waters, 1997 [84]
	Deterioration of temporal memory for recall of faces after 36 h of sleep deprivation	Harrison and Horne, 2000 [85]
Mood	Suffer negative moods	Dinges et al., 1997 [86]
Thinking	Sleep loss produces temporary changes in cerebral metabolism, cognition, emotion, and behavior consistent with mild prefrontal lobe dysfunction	Killgore et al., 2008 [87]
Learning and academic performance	Poor declarative and procedural learning in students, but once sleep was optimized, improvement in academic performance noted	Curcio et al., 2006 [88]
Vigilance	Feedback blunting could be caused by general, downstream impairments from loss of vigilant attention due to sleep deprivation	Lim and Dinges, 2010 [89]
Reaction time	Greater subjective reliance on avoidance as a coping strategy was associated with greater deterioration in performance	Saadat et al., 2017 [90]
Personal injury	Decreased duration led to increased work-related injury	Lombardi et al., 2010 [91]
Traffic accidents	Increased risk of traffic accidents	de Mello et al., 2013 [92]; Pandi-Perumal et al., 2006 [93]
	Increased traffic accidents among doctors under study	Steele et al., 1999 [94]; Schlafer et al., 2014 [66]
	More likely to be involved in vehicular crashes	Drake et al., 2010 [95]
	Increased auto accidents	Coren, 1996 [96]
	Increased car accidents among adolescents	National sleep foundation, 2006 [97]
Industrial accidents	Increased workplace injuries	Barnes and Wagner, 2009 [98]; Lahti et al., 2006 [99]; Kantermann et al., 2007 [100]
Medical errors	Sleep disturbances and internship-enforced short sleep increased risk of depression development and chronicity and medical errors	Kalmbach et al. 2017 [101]
Decision making	Deterioration in decision-making	Linde et.al., 1999 [102]
	Less effective in making decisions	Killgore et al., 2006 [103]
	Affects decision-making	Harrison, 2000 [85]
Moral judgement	Moral reasoning was severely impaired during partial sleep deprivation	Olsen et. al., 2010 [104]
	Lack of sleep is associated with low moral awareness	Christopher Barnes et al., 2014 [105]
